# Experience with the management of anorectal malformations in Ibadan, Nigeria

**DOI:** 10.11604/pamj.2021.38.214.21690

**Published:** 2021-02-24

**Authors:** Olakayode Olaolu Ogundoyin, Dare Isaac Olulana, Taiwo Akeem Lawal

**Affiliations:** 1 Department of Surgery, College of Medicine, University of Ibadan, Ibadan, Nigeria

**Keywords:** Associated anomalies, challenges, follow-up, management outcome

## Abstract

**Introduction:**

anorectal malformation is a multi-systemic birth defect of the distal gastrointestinal tract, the management of which is challenging to the surgeons, the patients and the parents. The presence of associated congenital malformations may worsen the outcome with consequent psychosocial effects on the patients and the parents. The characteristics of anorectal malformations with the challenges associated with their management and the outcomes are therefore presented here.

**Methods:**

all patients managed for anorectal malformations from January 2003 to December 2017 were studied. Patients´ demography, clinical presentations, types of malformations, associated anomalies, procedures performed, post-operative complications and management outcome were obtained and analysed.

**Results:**

eighty-eight children with anorectal malformations comprising 61 (69.3%) boys and 27 (30.7%) girls were studied with 76 (86.3%) patients presenting within the first year of life. Low anorectal malformation was observed in 14 (15.9%) patients, 71 (80.7%) patients had intermediate or high malformations and cloacal malformation was present in 3 (3.4%) patients. Associated congenital malformations were observed in 18 (20.5%) patients with 10 (55.6%) patients associated with intermediate or high malformations and urogenital system was the most common system whose anomalies were associated with anorectal malformations in 12 (13.6%) patients. Anoplasty was performed on 14 (15.9%) patients, posterior sagittal anorectoplasty was performed on 67 (76.1%) patients, abdominosacroperineal pull through on 4 (4.6%) patients and posterior sagittal anorectovaginourethroplasty on 3 (3.4%) patients. Six (6.8%) neonates died.

**Conclusion:**

immediate post-operative outcome was good; however, good functional outcome can only be assessed in an atmosphere of good follow-up which is still a problem in our environment.

## Introduction

Anorectal malformation (ARM) is a common anomaly that results from defective development of the distal gastrointestinal tract affecting both sexes, the management of which is challenging [1, 2]. There is a geographical variation in the incidence of ARM but it is reported as 1 in 5,000 live births in developed countries and more common in boys than girls [1, 3]. ARM is the commonest cause of intestinal obstruction in neonates [4, 5] and the commonest congenital anomaly causing intestinal obstruction in the older children [6]. Of the 151 cases of neonatal intestinal obstruction studied by Ameh and Chirdan [4], ARM accounted for 68.9% and 39.4% of 71 cases studied by Osifo and Okolo [5] in different parts of Nigeria. In the older children, it also accounted for 22.3% of 213 cases of intestinal obstruction studied by Ogundoyin *et al*. [6].

It is a multi-systemic birth defect which affects boys and girls involving the distal part of the gastrointestinal and genitourinary systems. As a result of this association, patients suffering from anorectal malformation tend to have faecal and urinary incontinence. This problem may persist even after being attended to by a paediatric surgeon with consequent psychosocial effects on the children and their parents [7, 8]. They could even have long term challenges like sexual dysfunction and reproductive difficulty. This is mostly because of the presence and magnitude of the associated anomalies in such patients and the challenges associated with the operative management of these anomalies [3].

Imperforate anus as it is sometimes called does not really and truly depict the nature of this congenital anomaly (ARM). A typical patient with ARM would most likely have an imperforate anus, with the distal enteric component ending blindly (atresia) or in form of a fistula into the perineum, urinary and genital tracts. Thus, there are three different types of ARM. These include low ARM whereby the rectal pouch is located below the level of the puborectalis muscle regardless of its association with a fistula. Whereas the intermediate and high ARMs are those whereby the rectal pouch either lies at or above the level of the puborectalis muscle [9]. This study presents the characteristics of ARM with the challenges associated with their management and the outcomes in our environment.

## Methods

All patients managed for ARM in the Division of Paediatric Surgery of the University College Hospital, Ibadan, Nigeria from January 2003 to December 2017 were studied. Following ethical approval by UI/UCH Institutional Ethical Review Committee, information about the patients´ demography, clinical presentations, types of malformations, associated anomalies, procedures performed, post-operative complications and management outcome were obtained from their medical records. Their initial management included resuscitation for all patients and additional colostomy for the patients with intermediate and high ARMs. Definitive management included anoplasty for patients with low malformations, posterior sagittal anorectoplasty (PSARP) for intermediate or high malformations, abdominosacroperineal pull through (Stephen´s procedure) for the very high malformations and posterior sagittal anorectovaginourethroplasty (PSARVUP) for cloacal malformations. Data obtained were recorded and analysed using IBM Statistical Package for Social Sciences (SPSS) Version 23.

## Results

A total of 88 children with ARM comprising 61 (69.3%) boys and 27 (30.7%) girls (M:F-2: 1) were studied. The age at presentation ranged from 1 day to 8 years with a median of 4.5 days. Seventy-six (86.3%) patients presented within the first year of life. Of these, 59 (67.1%) were neonates and only 6 (6.8%) presented after the age of 5 years (Table 1). Of the 59 neonates, 19 (32.2%) neonates presented within the first 48 hours of life and this rose to 37 (62.7%) neonates after 72 hours of life. The major clinical presentations included absent anus in 38 (43.2%) patients, failure/inability to pass stool in 40 (45.5%) patients and 25 (27.3%) patients had abdominal distension. Other presentations were fever in 4 (4.5%) patients, vomiting in 2 (2.2%) and anal stenosis in 1 (1.1%) patient. Low ARM was observed in 14 (15.9%) patients, 71 (80.7%) patients had either intermediate or high malformations and cloacal malformation was present in 3 (3.4%) patients.

**Table 1:** age and sex distribution of the patients

Grouped age	Sex		Total (%)
	Male (%)	Female (%)	
< 1 Month	43 (72.9)	16 (27.1)	59 (67.1)
1 Month – 1 Year	10 (58.8)	7 (41.2)	17 (19.3)
2 – 5 Years	4 (66.7)	2 (33.3)	6 (6.8)
> 5 Years	4 (66.7)	2 (33.3)	6 (6.8)
Total	61 (69.3)	27 (30.7)	88 (100.0)

Associated congenital malformations were observed in 18 (20.5%) patients with 2 (2.3%) patients having more than one associated anomalies. Twelve (66.7%) patients were associated with intermediate or high malformations and 6 (33.3%) were associated with low malformations. The urogenital system was the most common system whose anomalies were associated with ARM in 12 (13.6%) patients (Table 2). Eleven (12.5%) neonates managed in the last three years of the study had perineal ultrasonography to determine the type of anorectal malformations they presented with. Diagnosis of high ARM was made on 6 (6.8%) patients, intermediate ARM on 3 (3.4%) patients and low ARM on 1 (1.1%) patient but diagnosis could not be made on 1 (1.1%) patient as the distal pouch was severely dilated and the muscles could not be identified properly. The perineal ultrasonographic findings were, however, confirmatory for all the neonates.

**Table 2:** associated congenital malformations

Anomalies	Low ARM	High ARM
Congenital heart defects	0	3
Oesophageal atresia with tracheo-oesophageal fistula	0	2
Situs inversus	1	0
Jejuno-ileal atresia	0	1
Malrotation	0	1
Umbilical hernia	1	0
Inguinoscrotal hernia	2	3
Undescended testis	0	1
Hypospadias	1	2
Bifid scrotum	1	0
Septate uterus	0	1
Vaginal atresia	0	1

Anoplasty was performed on 14 (15.9%) patients, PSARP was performed on 67 (76.1%) patients, abdominosacroperineal pull through on 4 (4.6%) patients and PSARVUP on 3 (3.4%) patients. Forty-four patients (50%) had ARM without a fistula, however, recto-urethral fistula and recto-vestibular fistula were observed in 19 patients (21.6%) each whereas ano-cutaneous and ano-vestibular fistulas were observed in 1 patient (1.1%) each. Overall, 6 (6.8%) neonates with intermediate/high malformations died (Table 3). Also, mortality was observed in 5 (83.3%) boys and 1 (16.7%) girl (Table 4). The causes of death included prematurity, associated multiple congenital anomalies, respiratory distress and sepsis.

**Table 3:** relationship between patients´ age and management outcome

Grouped age	Outcome			Total (%)
	Alive (%)	Dead (%)	DAMA (%)	
< 1 Month	52 (88.1)	6 (10.2)	1 (1.7)	59 (67.1)
1 Month - 1 Year	17 (100.0)	0 (0.0)	0 (0.0)	17 (19.3)
2 - 5 Years	6 (100.0)	0 (0.0)	0 (0.0)	6 (6.8)
> 5 Years	6 (100.0)	0 (0.0)	0 (0.0)	6 (6.8)
Total	81 (92.1)	6 (6.8)	1 (1.1)	88 (100.0)

DAMA-Discharged against medical advice

**Table 4:** the relationship of gender to management outcome

Outcome	Sex		Total (%)
	Male (%)	Female (%)	
Alive	54 (66.7)	27 (33.3)	81 (92.1)
Dead	5 (83.3)	1 (16.7)	6 (6.8)
DAMA	1 (100.0)	0 (0.0)	1 (1.1)
Total	60 (68.2)	28 (31.8)	88 (100.0)

There were ten post-operative complications observed in 19 (21.6%) patients. Of these, perineal wound dehiscence was the most common complication observed in 4 (4.6%) patients; anal mucosal prolapse in 3 (3.4%); 2 (2.3%) patients each had anal stenosis, faecal soiling, urethral injury and faecal incontinence while 1 (1.1%) had respiratory distress and perforation of the vagina. The immediate post-operative Kelly continent score for the patients with faecal soiling was 3 which later improved to 5. The median duration of follow-up was 2.50±1.05 months with a range of 1 month to 5years. None of these patients however showed features of lower urinary tract dysfunction (LUTD), although majority of them did not come for adequate follow-up to enable us diagnose LUTD.

## Discussion

ARM is a congenital anomaly whose psychosocial effect on the children and their parents is enormous even after treatment. The diagnosis is often clinical with early presentation in majority of the patients. The absence of an anus at birth with consequent inability to pass meconium and abdominal distension are symptoms that often make majority of the patients to present in the hospital within the first 72 hours of life [10]. However, the presence of an associated fistula that allows for escape of meconium may delay presentations in some other patients. Thus, the age at presentation was similar with findings from previous reports [11, 12], but the sex presentation contradicts the reports of Sowande *et al*. [2] and Archibong and Idika [10] from the same region.

Expectedly, failure or inability to pass meconium and absent anus were the commonest clinical presentations prompting them to present early. However, vomiting and fever are relatively infrequent presentations that may suggest presence of complications like enterocolitis and upper urinary tract infection. Late presentation is usually occasioned by ignorance and unsupervised delivery at home including the fact that majority of the patients live in the rural areas and people have to pay out of pocket for medical care in this environment. The incidence of associated congenital malformation varies from 50% to 60%, they are often multisystemic and predominantly involving the skeletal and urogenital systems [9, 10]. This study revealed a 20.5% incidence of associated congenital malformations observed more in patients with intermediate/high ARM and urogenital system was the most common associated anomaly. The high default rate on discharge after surgery which often do not allow for adequate assessment of these patients for associated anomalies as suggested by Adejuyigbe *et al*. [11] may be responsible for the low incidence of associated anomalies in this series.

The most common type of malformations observed in this series are the intermediate/high anomalies which is in consonance with earlier reports [10-12]. The fact that paediatric surgeons are not many in this country and adult general surgeons who may be more familiar and comfortable with the relatively easier procedures for the management of low malformations in comparison with the more challenging PSARP (for intermediate/high malformations) are involved in the care of patients with low malformations in the peripheral hospitals may account for the relatively fewer numbers of low malformations and low incidence of associated malformations observed. Although the diagnosis of ARM is clinical, imaging studies are important in the management of ARM to determine the type of malformation and to diagnose presence of associated anomalies.

The traditional lateral prone transtrochanteric X-ray (cross table radiography) is valuable in the diagnosis of ARM without a fistula. The use of contrast studies may prove valuable in evaluating the anatomy of the fistula. Ultrasonography is also valuable in the diagnosis of associated anomalies and the determination of the type of ARM. Thus, the sonographic approaches in the determination of the type of malformation include suprapubic, perineal and infracoccygeal approaches [13]. These approaches are valuable in determining the distance between the rectal pouch and the perineum (pouch-perineal distance) [13], identification of the puborectalis muscle, the presence of a fistula [14, 15] and are devoid of ionising radiation. The perineal approach was used in this study because it has the advantages of determining the pouch-perineal distance and identifying an associated fistula.

The preliminary results revealed that perineal ultrasonographic findings correlated well with the findings of cross table radiography, contrast studies and the operative findings. Thus, confirming earlier reports on the validity of perineal ultrasound in the determination of the type of anorectal malformations [16, 17]. Staged repair of high ARM comprising establishment of a colostomy, the PSARP and closure of colostomy was adopted in this series as earlier suggested by Devries and Pena [18]. The colostomy is often performed in the neonatal period while PSARP and closure of colostomy would be performed 4-6 months later. This is to allow for adequate care of colostomy and its complications and to allow for the parents to gather enough funds for the definitive surgery as majority of the patients usually pay out of pocket in our centre. A practice that is common to many developing countries [10, 19]. Although, primary neonatal repair has been variously reported with good outcome in the developed countries [20-23], staged repair is still very relevant and safe in the developing countries where late presentation, sepsis and presence of associated severe malformations are daunting challenges that may affect the management outcome. But the morbidity associated with management of colostomy may also affect outcome of staged repair [20].

Functional constipation has been reported to be the most common complication following operative management of high ARM [24], this was not observed in this series as majority of the patients did not attend follow-up clinic sufficiently enough to discover this complication. The overall complication rate in this series is low in comparison with earlier reports [2, 11] with wound related complications more frequently encountered. Other significant complications like mucosal prolapse, faecal soiling and faecal incontinence were observed in patients with high malformations who had PSARP and abdominosacroperineal pull through. The two patients with faecal soiling improved during follow-up. The follow-up evaluation of the patients for continence and LUTD was difficult in this series as majority of the patients defaulted after discharge. This may be responsible for the relatively low incidence of faecal incontinence and absence of LUTD symptoms observed. Various scoring systems have been used to assess continence following PSARP with varying results [25], the Kelly scoring system was adopted in this series for the few patients that came for follow-up and the functional outcome was good. Mortality was observed only in the neonates and the causes of death were not surgically related.

## Conclusion

The management of ARM has evolved tremendously in our environment with good postoperative outcome despite its challenges. The functional outcome of repair of intermediate/high malformations in terms of maintenance of faecal continence, absence of faecal soiling and LUTD still poses some dire psychosocial consequences to the patients and their parents because of the poor follow-up culture in our environment. This can only be reduced if the parents are properly educated on the need to come for regular post-operative evaluation and efforts made by the health care givers to locate defaulting patients before it is too late.

### What is known about this topic

- Epidemiology of anorectal malformations in terms of the age and sex at presentation as well as variation in worldwide incidence of anorectal malformations;
- Associated congenital anomalies in which skeletal, genital and urinary anomalies are the commonest.

### What this study adds

- Variation in the sex incidence of anorectal malformations in which the male sex was more involved (M:F-2:1) compared to previous findings (M:F-1:1.3 and 1:1.5) in the same region of the country;
- The use of ultrasonography to determine the type of anorectal malformation, the anatomy of the fistula and the state of the puborectalis muscle which is cheaper and devoid of ionising radiation compared to the combined use of lateral prone transtrochanteric X-ray and contrast study to get the same result;
- Reduction in the incidence of associated congenital malformations compared to previous studies.
